# The United States Food and Drug Administration’s Platform Technology Designation to Expedite the Development of Drugs

**DOI:** 10.3390/pharmaceutics16070918

**Published:** 2024-07-10

**Authors:** Sarfaraz K. Niazi

**Affiliations:** College of Pharmacy, University of Illinois, Chicago, IL 60612, USA; niazi@niazi.com; Tel.: +1-312-297-0000

**Keywords:** FDA, platform technology, RNA, recombinant, regulatory approval

## Abstract

Drug development costs can be significantly reduced if proven “platform” technologies are allowed to be used without having to validate their use. The most recent US Food and Drug Administration (FDA) guideline brings more clarity, as well as a greater focus on the most complex technologies that can now be used for faster drug development. The FDA has highlights the use of lipid nanoparticles (LNPs) to package and deliver mRNA vaccines, gene therapy, and short (2–20 length) synthetic nucleotides (siRNA). Additionally, monoclonal antibody cell development is targeted. The FDA provides a systematic process of requesting platform status to benefit from its advantages. It brings advanced science and rationality into regulatory steps for the FDA’s approval of drugs and biologicals.

## 1. Background

Drug development platform technology refers to an array of foundational tools, methodologies, and systems designed to facilitate the efficient creation and production of pharmaceutical drugs. These technologies are integral to streamlining various stages of drug development, ranging from discovery and preclinical testing to clinical trials and manufacturing. Central to this approach are modular methodologies that incorporate standardized processes and reusability. Employing well-defined, replicable processes across multiple projects and adapting technologies developed for one drug to others can significantly reduce the time requirements and cost of drug development [1].

High-throughput screening (HTS) exemplifies these technologies, leveraging automation and robotics to screen vast libraries of compounds for potential therapeutic activity. Integrating bioinformatics tools allows for rapid analysis of biological data, enhancing the efficiency of the screening process. Advances in genomics and proteomics, such as next-generation sequencing (NGS) and proteomic technologies, play a crucial role by enabling the rapid sequencing of genomes and the study of proteins and their functions and interactions. These insights are vital for understanding disease mechanisms and identifying drug targets [2].

Computational drug design is another cornerstone of platform technology, utilizing in silico modeling to predict drug–target interactions and optimize drug candidates. Artificial intelligence (AI) and machine learning further augment these capabilities by predicting outcomes and refining drug development processes. Biomanufacturing platforms, which include advanced cell culture technologies and upstream and downstream processing techniques, are essential for producing and purifying drugs at scale. These platforms ensure the consistent and high-quality production of biologics [3].

Formulation and delivery systems represent another critical aspect of platform technology. Innovations such as nanotechnology and drug encapsulation enhance the stability and bioavailability of drugs, enabling targeted delivery and improved therapeutic outcomes [4].

Clinical trial innovations, including adaptive trial designs and the use of real-world data (RWD) and real-world evidence (RWE), provide flexible and comprehensive approaches to assessing drug efficacy and safety. These methodologies allow for modifications based on interim results and the incorporation of data from real-world settings, complementing traditional clinical trials [5].

Biomarker development is also integral to platform technology, with diagnostic biomarkers predicting responses to therapy and prognostic biomarkers forecasting disease progression. These biomarkers enable personalized medicine approaches, improving patient outcomes by tailoring treatments to individual needs [6].

Prominent examples of platform technologies in drug development include mRNA technology, CRISPR-Cas9, monoclonal antibody platforms, and CAR-T cell therapy platforms. The mRNA technology used in COVID-19 vaccines by Pfizer-BioNTech and Moderna demonstrates the rapid adaptability of this platform for various infectious diseases [7].

CRISPR-Cas9, a genome-editing technology, has transformative potential in creating targeted gene therapies [8].

New drug delivery systems lead to new drug effects, improved safety and accuracy, and new therapies for rare diseases. The combination of herbal extracts and essential oils with click chemistry can lead to the development of safer treatments for rare diseases like Duchenne muscular dystrophy [9].

Monoclonal antibody platforms streamline the production and optimization of antibodies for numerous diseases [10], while CAR-T cell therapy platforms offer personalized cancer treatments by engineering patients’ T cells to target cancer cells [11].

The regulatory landscape also plays a pivotal role in developing platform technologies. Regulatory bodies such as the EMA [12] have provided standardized guidelines and frameworks to ensure the safety, efficacy, and quality of drugs developed using these technologies. These guidelines facilitate the regulatory approval process, making it more predictable and efficient.

However, the FDA issued the most significant guideline in May 2024 [13], outlining the process and criteria for designating platform technologies in drug development. This program, established by section 506K of the Federal Food, Drug, and Cosmetic Act (FD&C Act), aims to enhance efficiencies in developing, manufacturing, and reviewing drug products incorporating designated platform technologies. The guidance provides detailed criteria for eligibility, the potential benefits of receiving a designation, and the procedural steps for requesting and maintaining a designation. The definitions of the terms used in this guidance document are presented as follows:

- Designated platform technology: A technology that meets the eligibility factors for designation under section 506K(b), (d), and (h) of the FD&C Act;
- Platform technology: A well-understood and reproducible technology essential to a drug’s structure or function, adaptable for multiple drugs, and facilitating standardized production or manufacturing processes;
- Preliminary evidence: Information from tests or studies comparing the platform technology’s use in approved and proposed drugs;
- Prior knowledge: Expertise and understanding gained from developing similar products and processes, including established scientific principles;
- Significant efficiencies: Leveraging previous tests or processes to streamline drug development, manufacturing, and review;
- Drug: For this guidance, the terms *drug*, *drug product*, and *product* refer to a drug as defined in section 201(g)(1) of the FD&C Act (21 U.S.C. 321(g)(1)). This includes biological products as defined in section 351(i) of the Public Health Service Act (PHS Act) (42 U.S.C. 262(i)). The term drug also applies to a drug or biological product constituent part (21 CFR 4.2) of a combination product being developed for review under section 505 of the FD&C Act (21 U.S.C. 355) or section 351 of the PHS Act.

## 2. Introduction

The FDA has recognized the potential of platform technologies to streamline drug development and has been actively involved in their promotion to reduce the burden on developers. One notable initiative is the Critical Path Initiative [14], launched in 2004, aimed at modernizing the scientific process through which drugs are developed, evaluated, and manufactured. This initiative encourages the adoption of innovative tools and methods, including platform technologies. Additionally, the FDA’s Breakthrough Therapy designation, introduced in 2012 [15], supports the accelerated development of drugs that substantially improve existing therapies for severe or life-threatening conditions. The FDA has also issued guidance on platform technologies, such as using master protocols in clinical trials to expedite the evaluation of therapies [16]. Furthermore, the FDA has supported using advanced manufacturing technologies, like continuous manufacturing, to ensure more efficient and reliable production processes [17]. These efforts collectively aim to enhance drug development and approval processes’ efficiency, predictability, and cost-effectiveness.

The FDA’s newly released Draft Guidance, Platform Technology Designation Program for Drug Development [13], issued in May 2024, pertains to Section 506K of the Federal Food, Drug, and Cosmetic Act (FD&C Act) (21 U.S.C. 356k) that was added by the PREVENT Pandemics Act, which, in turn, was enacted as part of the Consolidated Appropriations Act, 2023 (Public Law 117-328). This guideline elaborates on a new designation program for platform technologies. It intends to improve drug development, manufacturing, and application review processes incorporating designated platform technologies. 

The FDA emphasizes that potential benefits for sponsors receiving platform technology designation include receiving timely advice and having additional engagement with the FDA during product development, where the FDA may prioritize interactions if platform technology designation can have the most significant public health benefit or impact, leveraging data from prior approved products, such as batch or stability data, as well as nonclinical safety data to streamline nonclinical testing. 

The implementation of this guidance needed detailed advice from the FDA, including eligibility factors for receiving a platform technology designation, potential benefits of receiving such designation, how to leverage data from designated platform technologies, how to discuss a planned designation request as part of a milestone meeting, the recommended content of a designation request submission, and the review timelines for a designation request. 

This program is intended to result in efficiencies in drug development, manufacturing, and review processes for drug product applications that incorporate designated platform technologies. As a fresh start, it is possible that prior technologies that were previously considered may not retain this status, as new definitions must apply. With this disqualification, sponsors who had leveraged prior knowledge from previously submitted applications when authorizing or approving drugs in an application submitted by the same sponsor will be unable to capitalize on these concessions.

The FDA has redefined “platform” as something that is incorporated into or utilized by a drug or biologic and is essential to its structure or function; it can be adapted for, incorporated into, or utilized by more than one drug or biologic that shares common structural elements, and it facilitates the manufacture or development of more than one drug or biologic through a standardized production or manufacturing process or processes.

The criteria for designation are determined if it is incorporated in or used by an approved drug or biologic; its preliminary evidence demonstrates that the platform technology has the potential to be incorporated in or used by more than one drug or biologic without an adverse effect on quality, manufacturing, or safety; and its data or information indicate that incorporation or use of the platform technology has a reasonable likelihood of bringing significant efficiency.

While the scope of the guidance is not limited and its application is dependent on developers creating novel approaches to exploit the benefits of the program, the FDA identifies the following potential platform technologies: lipid nanoparticle (LNP) platforms for mRNA vaccines or gene therapy products, monoclonal antibody platform technologies, platforms that use a chemically defined targeting moiety conjugated with a well-characterized synthetic siRNA, and LNP platforms that encapsulate various short, single-stranded or double-stranded oligonucleotides. 

## 3. Potential Benefits of a Platform Technology Designation

Information about a designated platform technology may be leveraged in a subsequent application when supported by sufficient preliminary evidence. The application should be from the sponsor originally granted the platform technology designation. Alternatively, it can be from a sponsor with full reference rights to that information. Potential benefits to a sponsor that is granted a platform technology designation for a subsequent application generally include one or more of the following, as deemed appropriate by the FDA:

- Engaging in early interactions with the FDA to discuss using a platform technology, including information relevant to establishing, as applicable, safety, purity, potency, or quality.
- Receiving timely advice from and engaging with the FDA during the development program, such as additional interactions and/or meetings on the use of the platform technology. Depending on resources, the FDA might prioritize interactions or additional engagements regarding a designated platform technology for those products where the FDA has determined the most significant public health benefit or impact.
- Leveraging data from a prior product that used the designated platform technology, such as leveraging batch and stability data from a related product as prior knowledge that can supplement product development studies (e.g., in-use stability studies to define administration conditions and/or light exposure studies to inform the design of the container closure system) or support shelf-life extrapolation and determination for structurally alike products.
- Leveraging certain nonclinical safety data from prior products that used the designated platform technology such that a product-specific assessment for specific, designated endpoints might not be warranted.
- Considering previous inspection findings by the FDA for subsequent marketing applications related to the manufacture of a drug that incorporates or uses the designated platform technology.

Once a platform technology has received a designation, developing or assessing subsequent applications for drugs that use or incorporate the designated platform technology will not automatically be granted priority review based on using or incorporating a platform technology. The criteria for being granted a priority review are separate from this program. A platform technology designation does not affect product eligibility for expedited approval pathways if otherwise eligible.

## 4. Eligibility for the Platform Technology Designation Program

To determine eligibility for designation as a designated platform technology, the FDA will first determine whether the technology qualifies as a platform technology. Under section 506K(h)(1) of the FD&C Act, a platform technology is a well-understood and reproducible technology that may include a nucleic acid sequence, molecular structure, mechanism of action, delivery method, vector, or a combination of any such technologies that the FDA determines to be appropriate, where the sponsor demonstrates that the technology (1) is incorporated in or used by a drug or biological product and is essential to the structure or function of such drug or biological product; (2) can be adapted for, incorporated into, or used by more than one drug or biological product sharing common structural elements; and (3) facilitates the manufacture or development of more than one drug or biological product through a standardized production or manufacturing process or processes.

Under section 506K(b) of the FD&C Act, a platform technology incorporated within or used by a drug or biological product is eligible for designation as a designated platform technology by the FDA if (1) it is incorporated in or used by an approved drug (i.e., the FDA reviewed and approved an application for a product incorporating or using the platform technology); (2) preliminary evidence demonstrates that the platform technology has the potential to be incorporated in or used by more than one drug without an adverse effect on quality, manufacturing, or safety (the preliminary evidence would be submitted by the sponsor of the approved or licensed drug or by an applicant who has been granted a right of reference to data submitted in the application for such drug); and (3) data or information submitted by the applicable person indicate that incorporation or usage of the platform technology has a reasonable likelihood of bringing significant efficiencies to the drug development or manufacturing process and to the review process.

For this guidance, preliminary evidence, as referred to in section 506K(b)(2), means information from completed tests or studies comparing the platform technology used in the approved or licensed drug(s) with the proposed use of the platform technology in the drug(s) under investigation described in the designation request. To support a designation, this information must sufficiently demonstrate the potential for the platform technology to be incorporated in or used by the drug(s) under investigation without adversely affecting quality, manufacturing, or safety. For example, suppose the sponsor wants to leverage stability testing. In that case, the preliminary evidence should demonstrate the similarities in the molecule and manufacturing process to justify leveraging stability data. In addition to the same manufacturing process, to ensure consistency and mitigate unanticipated minor differences that could result in differences in product performance and safety, drug product manufacturing should generally occur at the same manufacturing site. For a proposed manufacturing site change, the FDA may ask for additional quality data, e.g., stability data, as a bridge between different manufacturing sites.

There should be minimal differences between the approved or licensed drug(s) using the platform technology and the drug(s) under investigation as part of an IND application that proposes to use the same platform technology. Such information could involve establishing minimal differences in structure, mechanism of action, biological effects, or manufacturing processes that could affect quality or safety. Preliminary evidence should also consider what information the applicant proposes to leverage. Preliminary evidence could include but is not limited to information on the following:

- Structurally similar drug substances, such as similarly sized nucleic acid sequences with comparable backbone chemistry, subunit modifications, and targeting moieties;
- Minimal qualitative and quantitative differences in drug product formulation; and/or
- Nearly identical manufacturing processes for drug substance and/or drug product manufacturing and purification.

As part of establishing preliminary evidence, the requester should include in their assessment all the products that use or incorporate the platform technology, regardless of current developmental or marketing status. The designation request should consist of summary data from the evaluations of all such products. The requester should include an adequate justification explaining why the summary data are sufficient to show that specific product-specific tests, analyses, or studies can be leveraged.

For the purposes of the platform technology designation program, significant efficiencies in the drug development or manufacturing process and the review process mean that a prior test, study, or manufacturing process involving the approved or licensed drug described in section 506(K)(b)(1) of the FD&C Act could be leveraged in a subsequent application in such a way as to allow the subsequent application incorporating such information to be developed and reviewed in a generally more streamlined manner. Summary evidence from completed studies should be submitted to demonstrate that there is a reasonable likelihood that significant efficiencies exist.

## 5. Designation Request

A request for the designation of a platform technology should be made at the investigational new drug (IND) stage of development for a planned subsequent new drug application (NDA), 351(a) biologics license application (BLA), or request for Emergency Use Authorization. This request should include a clear argument, including summary data from completed studies, demonstrating that incorporating or utilizing the platform technology is fairly anticipated to result in significant efficiencies in the review process. The preliminary evidence consists of data and findings from completed tests or studies that evaluate the proposed use of the platform technology in an already approved drug product, the proposed use of the same platform technology in a new drug product, or the use of a platform technology across scenarios (making comparisons between scenarios). There should be “minimal differences” between the approved product that employs the platform technology and the product under investigation that seeks to use the same technology. A designation request should also include the following content: identification of a shared structural element between drug products; justification and scientific support for the use of a platform technology across products, noting how subsequent uses would not affect safety, quality, or manufacturing; and a risk assessment evaluating how later uses of the platform technology might affect the relevance of the prior information. 

The designation does not grant third parties additional rights of reference to platform technology information unless they own or have full rights to that information; any third party wishing to leverage information provided in a prior application related to the designated platform technology should submit an agreement demonstrating a full right of reference in Module 1. A BLA holder must have knowledge of and control over its product’s manufacturing process; NDA sponsors can use platform technology information from previous applications by cross-referencing, but BLA sponsors must include all information in future applications. 

It may take actions to expedite the development and review of applications that incorporate designated platform technologies. However, this does not result in an expedited action date clock under the FDA’s established user-fee timelines, nor does it affect eligibility for expedited approval pathways. The FDA will decide within 90 days after receiving designation requests; however, a finding of ineligibility does not preclude the use of previous knowledge across applications submitted by the same sponsor. Meetings to discuss the prospect of platform technology designation can be requested following the FDA’s formal meetings. Between the FDA and PDUFA Product Sponsors or Applicants, draft guidance and designation requests should be presented concurrently with the original filing of an IND or as an update. 

### 5.1. Recommended Content for a Designation Request

A submission requesting that FDA grant a platform technology designation should include the following:

- Specifically, the request should explain how the technology meets the definition under 506K(h)(1) and how it is eligible under 506K(b).
- Identification of an approved application (NDA, BLA, or ANDA) where the technology was incorporated, with applicable cross-references to other applications or submissions that the sponsor owns or has full right of reference to as part of a business agreement, and an appropriate eCTD link to the relevant and identified information (in INDs, NDAs, BLAs, or ANDAs).
- Identification of the shared structural element between drug products and how the shared structural element facilitates the use of the platform technology. Such a demonstration for a shared element could be based on a logical assertion supported using relevant prior knowledge and/or experimental studies.
- Justification and scientific support for using a platform technology across multiple drugs, including how utilizing the technology in subsequent proposed products would not affect safety, quality, or manufacturing. The justification should include information to demonstrate, for example, how the technology can be incorporated into other drugs with no or only minor differences in the relevant parts of the manufacturing process, as well as how the technology functions and is appropriate for the safety and quality profile.
- Risk assessment to evaluate how the differences between a prior product and the subsequent proposed product (for this guidance, a *subsequent proposed product* is a proposed drug product that is the subject of a marketing application and/or a candidate product that is the subject of an IND application) could affect the use of the platform technology; the relevance of prior information; and, therefore, how much prior information would be appropriate to be leveraged in support of the subsequent proposed product.
- Information to justify why the use of the platform technology would bring significant efficiencies to the drug development or manufacturing process and to the review process for the application (e.g., allow for testing or validation performed as part of the development of one of the products to reduce testing or validation for the other products and, thus, increase efficiency). The ability to reduce testing and validation for manufacturing and/or analytical methods will depend on the drug class. Whether the reduction in testing or validation constitutes a significant efficiency would depend on the nature of the testing or validation.

The above information should be described with sufficient detail to support an evaluation of the risks associated with leveraging information about the platform technology. The sponsor should clearly explain what data or information from the designated platform technology they propose to leverage. When specifying the data, studies, or other information from the designated platform technology to be leveraged in the subsequent proposed product, the sponsor should include an adequate justification explaining why this can be leveraged, where, otherwise, the sponsor might conduct specific tests for the following proposed product.

For attributes related to the product differences, the sponsor should provide developmental data or prior knowledge that address potential failure modes and consider proposals to address residual risk at the time of the initial filing of the application (e.g., additional specification tests, in-process controls, a higher number of in-process parameters, or narrower ranges for critical process parameters).

No other structural elements in the subsequent proposed product should interfere with the ability to leverage the development information on the prior product to support the subsequent proposed product (i.e., the sponsor should show how the platform technology can be used in the same way and to the same effect in the subsequent proposed product without other factors interfering).

There should also be no differences in manufacturing process parameters that would create uncertainty when leveraging manufacturing for the subsequent proposed product.

Although some minor differences in product design, operating conditions, and/or context of use might exist between products, the experience with the platform technology in one or more other products might allow for formulation and stability bracketing approaches to cover differences in operating conditions or contexts. Without cross-product experience, studies in relevant models can be used to expand the operating conditions or contexts to which a platform technology could be applied. If applicable, a comparison of raw material sources for products manufactured across a platform should be provided.

### 5.2. Submitting a Designation Request

Sponsors can request the designation of platform technology at any time concurrent with or after submitting an IND. Details of the electronic submission, formatting, and details of the document are provided in the guidance document.

Although a sponsor can request designation concurrent with the submission of an IND, the timing of the request for designation should consider whether there are adequate product-specific data. The FDA recommends the sponsor submit far enough into their development cycle to permit a determination of suitability for platform technology designation (e.g., whether the platform technology has the potential to be incorporated in or used by more than one drug without an adverse effect on quality, manufacturing, or safety). In most cases, this would likely be after a safe-to-proceed decision for the IND. The sponsor’s submission should indicate in the administrative documents in Module 1 that it is a request for a platform technology designation. 

### 5.3. Timing of Designation Request Submissions by the Requester and Timeline for FDA Evaluation of Designation Requests

A sponsor with an approved NDA or BLA that incorporates or uses the platform technology can submit a request for a platform technology designation concurrent with or at any time after the submission of an IND application (this can be done under either section 505(i) of the FD&C Act or section 351(a)(3) of the PHS Act for a subsequent drug product). The timing of the request for designation should consider whether adequate product-specific data are available for the prior and subsequent products. Although a sponsor can request designation concurrent with the submission of an IND, the FDA recommends that the sponsor submit far enough into the product’s development cycle to permit a determination of suitability for platform technology designation. In most cases, this would likely be after a safe-to-proceed decision for the IND. Any designation requests that are submitted at the same time as a new IND or a subsequent IND amendment will be evaluated separately from the safety assessment of the new IND or of any subsequent IND amendments (e.g., a proposed new clinical protocol or a Chemistry, Manufacturing, and Controls (CMC) or pharmacology/toxicology amendment). If the IND is placed on a full clinical hold, a simultaneously submitted designation request will be deemed inadequate for review. The FDA will determine whether the designation meets the eligibility factors and if the platform technology will be designated within 90 calendar days from receipt of the platform technology designation request. The FDA will provide a written explanation to the requester regarding the determination. 

## 6. Post-Approval Changes to a Designated Platform Technology

A sponsor can submit changes to an approved application that incorporates the designated platform technology via a post-approval supplement to the application. The supplement should be submitted following 21 CFR 314.70 or 601.12, as described by appropriate post-approval change guidance [18].

## 7. Revocation of a Platform Technology Designation

After a platform technology designation is granted, the FDA may revoke the designation if the FDA determines that the sponsor’s designated platform technology no longer meets the eligibility factors for the platform technology designation program. The FDA will communicate this revocation in writing, along with the rationale for the revocation.

The FDA may remove platform technology designations if eligibility conditions are no longer met; the sponsor will be informed of the revocation in writing, along with the FDA’s revocation rationale. Post-approval manufacturing supplements can be used to seek approval for changes to platform technologies. A single submission of grouped supplements can be utilized by a sponsor of multiple approved applications utilizing the platform technology; in advance of a planned change to a designated platform technology, an original application or a prior approval supplement can include one or more comparability protocols. 

## 8. General Considerations for Eligibility

The guideline is specific to the format of the request and the procedural steps. It provides significant teaching of the steps that a developer can take to request the designation of platform technology, as well as the intent to use the benefits of the designation. While it is generally expected at the IND level meeting, the FDA will accept such requests at any time, since these considerations may arrive at different stages of development.

Below are examples of potential platform technologies, with examples of key elements of each technology.

### 8.1. LNP Platforms

Lipid nanoparticle (LNP) platforms for mRNA vaccine or gene therapy products: (Although this example includes mRNA vaccine or gene therapy products, this is not intended to suggest that other cell or gene therapy products are inappropriate for the designation program.)

a. Composition, including type, amount, and manufacture of the lipids;
b. Manufacturing process unit operations (e.g., transcribing RNA, synthesizing lipid moieties, and formation of the lipid nanoparticles) that are not sensitive to inputs (e.g., template sequences) and yield consistent outputs across multiple products and where sequence differences of the mRNA do not affect product quality;
c. Manufacturing process parameters, in-process controls, and equipment critical to the manufacture of the mRNA LNP vaccine or gene therapy;
d. Process-related impurity clearance across a defined downstream purification process.

Lipid nanoparticle (LNP) technology was validated with the arrival of the COVID-19 mRNA vaccine delivered using LNP technology [19]. While this technology was critical in enabling the delivery of mRNA by protecting it from degradation and cellular entry, it has now become a therapeutic platform for many drugs [20,21], particularly the upcoming line of gene therapy products [22]. Classifying LNPs as a designated platform has many advantages for developers, as they can now select the types and amounts of lipids used in the formulations. Table 1 lists the LNP formulations used in products approved by the FDA. 

While these are not the only choices, copying this formulation will fall under the platform technology selection and significantly reduce the nonclinical toxicology testing requirements [23]. While there are many options, staying with a proven formulation of cationic ionizable lipid/helper phospholipid/cholesterol/PEG-lipid goes a long way in shortening the development cycle [24]. Ionizable lipids are essential for encapsulating mRNA and ensuring delivery into cells. Phospholipids help form the lipid bilayer structure, cholesterol provides structural integrity and fluidity, and polyethylene glycol (PEG) increases stability and circulation time. LNPs comprise several different types of lipids at varying ratios. For example, both Moderna’s Spikevax^®^ and Pfizer’s/BioNTech’s Comirnaty^®^ COVID-19 vaccines use LNP formulations consisting of four different lipids, namely an ionizable lipid, a neutral phospholipid (e.g., DPSC), cholesterol, and a PEG lipid (e.g., PEG-2000-DMG). 

Essential patents, including specific formulations, include the following:

US Patent 10,221,127: Covers ionizable lipids for delivering nucleic acids;

US Patent 9,504,651: Describes ionizable lipids that enhance nucleic acid delivery efficiency; 

US Patent 9,364,435: Covers compositions of LNPs and their use in providing mRNA;

US Patent 10,266,485: Describes methods for producing LNPs that encapsulate nucleic acids;

US Patent 9,364,435: Claims specific PEG-lipid conjugates that enhance the stability of LNPs;

US Patent 9,630,929: Describes PEG-lipids that can be used in LNP formulations to improve pharmacokinetic properties;

US Patent 10,758,599: Covers a specific formulation of LNPs for delivering mRNA;

US Patent 10,316,450: Describes a particular composition of lipids for LNPs that improves delivery efficiency.

While these hurdles appear challenging, there are many legal ways around these patents. First, patent information submission is not required when filing a Biologics License Application (BLA) with the FDA. Unlike the New Drug Application (NDA) process for small-molecule drugs, where patent information must be submitted and is listed in the Orange Book, the BLA process for biologics does not have an equivalent requirement for patent information submission or listing in a similar resource. However, the 351(k) BLA requires such a filing [25]. The FDA is not required to disclose the exact composition of LNPs, as it is considered proprietary information and is often protected as a trade secret by the developers. The responsibility of demonstrating infringement lies with the patent holder, which is generally a very complex process without knowledge about the composition. 

Second, patents can be worked around by changing the composition while keeping the components the same or using equivalent elements that are not patented. When using a different composition, it is advisable to discuss it with the FDA in meetings, as suggested above, if the proposed change will keep the platform status intact. 

**Figure 1:**
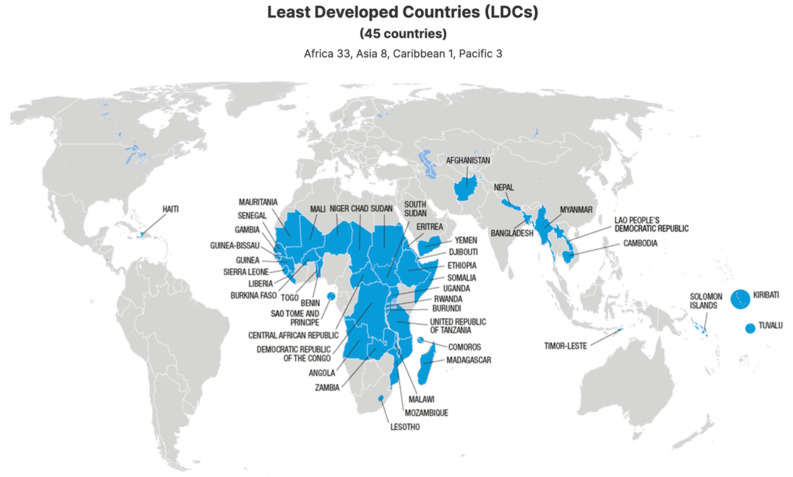
The Least Developed Countries where pharmaceutical and healthcare patents do not apply until 2034 [27].

Third, if the jurisdiction is within Least Developed Countries (LDCs) (Figure 1) as defined by the World Trade Organization and the United Nations, then none of these patents applies until 2034 [26].

While the composition of an LNP may be subject to intellectual property constraints, its manufacturing process involving RNA transcription, lipid synthesis, and nanoparticle formation yields consistent outputs, regardless of template sequences; however, these details may not be readily available to developers and, again, need discussion with the FDA. The critical process parameters and controls necessary for mRNA LNP production are widely available, especially as the US Pharmacopoeia [28] has published the release specifications and test methods that should be acceptable to the FDA. It is highly recommended that you follow these methods instead of creating your own to avoid validation issues and increase the chance of acceptance by the regulatory agencies. Finally, impurity clearance methods are well-defined, and the limits of impurities are published; these must be followed, and if there is any noncompliance, efforts should be made to fix the profile rather than justify any unknown impurity [29]. Differences in the number of impurities found in approved products can be justified but not the type of impurities.

Gene therapy products such as siRNA products are also delivered by LNPs [30,31] such as siRNA products. Two decades of research on RNA interference (RNAi) have resulted in a breakthrough discovery in biology, offering a robust platform for a new class of medicines that modulate mRNA expression. The FDA approved the first LNP-based genomic medicine in 2018, called Onpattro (siRNA patisiran), to treat polyneuropathy caused by hereditary transthyretin-mediated amyloidosis. It is a targeted RNA-based therapy that uses lipid nanoparticles (LNPs) to deliver siRNA directly into the liver. The siRNA then silences a portion of RNA that causes the disease, which alters or stops the production of disease-causing proteins

One consideration in developing LNP products is demonstrating that mRNA, siRNA, and other sequence changes do not impact product quality; this allows for maintenance of the platform status. 

Lipid nanoparticle platforms encapsulating different short, single-stranded, or double-stranded oligonucleotides include the following:

e. Composition, including type and amount of the lipids;
f. Demonstration that, within a narrow range of double-stranded or single-stranded oligonucleotide length, there is no effect on product quality arising from sequence differences of the oligonucleotides (product-specific stability data should be provided to demonstrate that the sequence changes, modifications to the sugar backbone, phosphorothioate incorporation, or nucleobase modifications of the single-stranded or double-stranded oligonucleotide will not impact product quality);
g. Manufacturing process parameters, in-process controls, and equipment critical to the formation of the lipid nanoparticles

### 8.2. mAbs

The FDA specifically mentions monoclonal antibodies (mAbs) and Fc-fusion proteins. These discussions also apply to other classes of therapeutic proteins amenable to platform approaches, including the following:

- Approaches for cell substrate and expression construct engineering that can be used with multiple products with the same upstream manufacturing process developed for the specific cell substrate and expression construct backbone refer to strategies in biotechnology and pharmaceutical manufacturing where a consistent and standardized upstream process is applied to produce various biological products using the same foundational cell substrate and expression construct [32].
- **Cell substrate engineering** involves selecting and genetically modifying a host cell line (cell substrate) to produce a desired biological product. Common host cell lines include Chinese hamster ovary (CHO) cells, human embryonic kidney (HEK) cells, and microbial cells like Escherichia coli [33]. For instance, CHO cells are frequently engineered to produce monoclonal antibodies (mAbs). By optimizing these cells to express high levels of recombinant proteins, manufacturers can use the same engineered CHO cell line to produce different mAbs, leveraging the same upstream process conditions, like media composition, growth conditions, and bioreactor settings [34]
- **Expression construct engineering** refers to the design and insertion of genetic constructs into the host cells to drive the expression of the target protein. The construct includes the gene of interest and regulatory elements such as promoters, enhancers, and terminators. For example, an expression construct might include a strong promoter like the cytomegalovirus (CMV) promoter to ensure high levels of protein expression in HEK293 cells. Once an effective expression construct backbone is developed and optimized, it can be reused with different genes of interest, simplifying the production of various recombinant proteins using the same upstream processes [35].
- Monoclonal Antibody Production:
  a. Cell substrate: CHO cells are engineered to produce various therapeutic monoclonal antibodies. The same upstream process, including cell culture media and bioreactor conditions, is applied across different mAbs, ensuring consistent product quality and streamlined regulatory approval [36].
  b. Expression construct: A well-characterized expression vector with a CMV promoter and other regulatory elements can be adapted to express different antibody genes, reducing development time for new products [37].
- Vaccine Manufacturing:
  a. Cell substrate: Vero cells (derived from African green monkey kidney cells) are used to produce viral vaccines like those for poliovirus and influenza. The upstream process, such as cell culture techniques and infection protocols, remains consistent across different vaccines produced in Vero cells [38].
  b. Expression construct: For recombinant protein vaccines, a plasmid vector with a strong viral promoter can be engineered to express different antigens, facilitating the rapid development of vaccines against emerging pathogens [39].
- Gene Therapy Products:
  a. Cell substrate: HEK293 cells are commonly used to produce viral vectors for gene therapy. The upstream process involving cell culture and transfection methods is standardized, enabling the production of various gene therapy vectors with the same manufacturing platform [39].
  b. Expression construct: The viral vector backbone, such as adeno-associated virus (AAV), is engineered with different therapeutic genes while keeping the regulatory elements constant, allowing for the efficient production of different gene therapy products using the same upstream process [40].

### 8.3. siRNA

Small interfering RNA (an oligonucleotide, a short sequence of nucleotides, typically consisting of 2 to 20 nucleotides (siRNA), the fundamental building blocks of DNA and RNA) is produced through both natural and synthetic methods. Naturally, siRNA is generated in cells as part of the RNA interference (RNAi) pathway, which regulates gene expression and defends against viral infections. The process begins with the enzyme Dicer, which recognizes and cleaves long double-stranded RNA (dsRNA) molecules into smaller fragments of about 20–25 base pairs, forming siRNA. These siRNA fragments are then incorporated into the RNA-induced silencing complex (RISC). Within RISC, the double-stranded siRNA is unwound into two single strands, namely the guide and passenger strands. The guide strand remains associated with RISC, guiding it to complementary mRNA molecules. Once bound to its target mRNA, RISC cleaves it, leading to its degradation and preventing translation into protein [41,42].

These molecules can be synthesized with a specific sequence, making them invaluable for various research, diagnostic, and therapy applications. In polymerase chain reaction (PCR) [43], oligonucleotides serve as primers to amplify specific DNA sequences, enabling the study and manipulation of genetic material [44]. They are also essential in DNA sequencing, acting as primers or probes to determine the order of nucleotides in a DNA molecule. In gene synthesis, oligonucleotides can be assembled into longer DNA or RNA sequences for the construction of genes and other genetic elements [45]. Additionally, oligonucleotides play a crucial role in antisense therapy by binding to specific mRNA sequences, thereby blocking gene translation in disease processes [46]. In the CRISPR-Cas9 genome-editing technology, oligonucleotides guide the Cas9 enzyme to particular DNA sequences, facilitating targeted gene editing [47]. Furthermore, oligonucleotides are used as diagnostic probes to detect specific DNA or RNA sequences in various diagnostic assays, enhancing the accuracy and efficiency of disease detection [48]. The versatility and specificity of oligonucleotides make them indispensable tools in molecular biology, with wide-ranging applications in scientific research and medical treatments.

Recombinant DNA technology and chemical synthesis of siRNA involve solid-phase synthesis, where siRNA is built nucleotide by nucleotide on a solid support using phosphoramidite chemistry. This method provides precise control over the sequence and allows for modifications to enhance stability and functionality. After synthesis, the siRNA is purified using high-performance liquid chromatography (HPLC) or other techniques to ensure high purity and the correct sequence [49]. Alternatively, siRNA can be produced through in vitro transcription. This method first generates a DNA template encoding the siRNA sequence utilizing PCR or cloning techniques. The template is then transcribed into RNA using RNA polymerase in an in vitro transcription reaction. The sense and antisense RNA strands are annealed to form the double-stranded siRNA and purified to remove impurities and byproducts [50].

Process-related impurity clearance is evaluated across a defined downstream purification process that can be used for multiple products with a minimal modification method; a plasmid or viral vector encoding the siRNA sequence is constructed and introduced into host cells such as bacteria, yeast, or mammalian cells. These host cells transcribe the siRNA sequence into RNA, which is then processed by the cellular machinery into functional siRNA. The siRNA is then extracted from the host cells and purified for use [51].

Platforms using a chemically defined targeting moiety in conjugation with a well-characterized synthetic siRNA include the following:

a. Identification of the targeting moiety, including its synthesis, incorporation into the final drug substance, and quality control;
b. Modification of the synthetic siRNA sequence has no biological effect on the product quality or safety arising from the differences such that some pharmacology/toxicology and CMC data are potentially appropriate to be leveraged;
c. The safety of the targeting moiety is not altered when used with multiple different siRNA moieties such that some pharmacology/toxicology data are potentially appropriate to be leveraged;
d. Use of a unique method of manufacturing, purification approach, or purification strategies that simplify downstream characterization of the drug product and that can be used for multiple products with little modification;
e. Recent advancements in delivery methods, such as the use of lipid nanoparticles, have further enhanced siRNA’s stability and cellular uptake, making it a more viable option for clinical applications [52].

## 9. Exclusions

For a technology to be designated, it must meet the platform technology definition in the statute and the eligibility factors for designation under 506(K)(b). Therefore, it is possible for a technology to meet the definition of a platform technology under 506K(h) but not be designated by the FDA as a designated platform technology. For example, a technology that meets the definition of a platform technology might be inappropriate for the designation program because current review processes already reflect the use of the well-understood technology or there is a public standard. Therefore, the FDA would not consider such technologies to meet the criterion of bringing significant efficiencies to the drug development, manufacturing, and review processes for the designated platform technology program. Examples of technologies that could be inappropriate for the designation program because the technologies do not meet the definition, criteria, or both include the following:

- Approaches to viral clearance for certain unit operations;
- Manufacturing unit operations sensitive to inputs (e.g., the general use of roller compaction that might be sensitive to material properties).
- Technologies that rely on established manufacturing unit operations (e.g., blending, compressing, or film coating) (this prior knowledge can already be leveraged in formulation and manufacturing process development; demonstrated prior knowledge can also be used in applications to demonstrate unit operation robustness).
- Established formulation technologies traditionally used for immediate-release and extended-release solid oral dosage forms (e.g., matrices and osmotic pumps), established formulation technologies for oral and parenteral dosage forms, and other established drug delivery systems;
- Near-infrared technologies for monitoring in-process material attributes;
- Analytical methods that leverage prior knowledge as described in the draft ICH guidance for industry *Q14 Analytical Procedure Development* (August 2022);
- Device delivery technologies (e.g., syringes and autoinjectors). (For the purposes of this guidance, generally, such device delivery technologies are not essential to the structure (e.g., chemical or molecular formula) or function (e.g., the molecular mechanism of action or the drug or biological product’s characteristics or chemical or biological interaction with the body) of the drug or biological product. In addition, such device delivery technologies are generally not expected to facilitate the manufacture or development of a drug because, generally, drug manufacture is complete before the drug interacts with the delivery device. Also, the devices are not expected to bring significant efficiencies to the review process because of the existing leveraging options for delivery devices already incorporated in the review process).

## 10. Conclusions

### 10.1. Biosimilars

Biological drugs with new indications do not meet the definition of biosimilars and are, thus, subjected to extensive testing as a new entity; one extension of platform technology that can be readily applied here is to allow for a hybrid approval if the technology employed in the latest biological drugs is the same. 

A significant debate is arising with respect to mRNA vaccines that are classified as biological drugs but present a chemical profile; allowing generic forms of these products is another consideration that the FDA can declare as platform technology and allow for “biosimilar mRNA products”. Can this concept be extended to CRISPR and gene therapy products?

Biosimilarity demonstration can be narrowed down if the plasmid structure used to express an antibody is similar or impurity profiling processes can be harmonized.

However, to benefit from the platform technology, the idea is to follow the process as used by an approved product; it may be difficult to secure details at times, but most developers should be able to use the FDA meeting provision in their favor by checking out their process validity.

### 10.2. Advanced Manufacturing

While modern manufacturing technologies such as continuous manufacturing, a process that allows for the uninterrupted production of pharmaceuticals, including biologics, rather than traditional batch methods, can be extended if the FDA would consider not requiring that a product with a new technology be approved to emulate the technology [17]. The FDA has approved products such as viral vectors for gene therapies that are produced using continuous manufacturing; extending this technology to other products should be a considered by the FDA.

### 10.3. Advanced Manufacturing Program

The FDA’s Office of Counterterrorism and Emerging Threats (OCET) promotes advanced manufacturing technologies, including continuous manufacturing. This program aims to enhance the supply chain’s resilience and improve the quality and consistency of pharmaceutical products [53]. The FDA’s Q13 guidance document outlines the scientific and regulatory considerations for developing, implementing, and managing continuous manufacturing processes for drug substances and products, including biologics.

## Figures and Tables

**Table 1:** Composition of FDA-approved LNP formulations of COVID-19 vaccines.

Type	Moderna	BioNtech-Pfizer	CureVac
Ionizable lipids	heptadecan-9-yl 8-((2-hydroxyethyl)(6-oxo-6-(undecyloxy)hexyl)amino)octanoate (SM-102)	(4-hydroxybutyl)azanediyl)bis(hexane-6,1-diyl)bis(2-hexyldecanoate) (ALC-0315)	Probably, (4-hydroxybutyl)azanediyl)bis(hexane-6,1-diyl)bis(2-hexyldecanoate) (ALC-0315)
Phospholipid	1,2-distearoyl-sn-glycero-3-phosphocholine (DSPC)	1,2-distearoyl-sn-glycero-3-phosphocholine (DSPC)	1,2-distearoyl-sn-glycero-3-phosphocholine (DSPC)
Cholesterol	Yes	Yes	Yes
PEG lipid	1,2-dimyristoylrac-glycero-3-methoxypolyethylene glycol-2000 (PEG2000 DMG)	2[(polyethylene glycol)-2000]-N,N-ditetradecylacetamide	Probably, 2[(polyethylene glycol)-2000]-N,N-ditetradecylacetamide
Total Lipids, mg/dose	1.93	0.77	0.31
